# Acupuncture for Infantile Colic: A Systematic Review of Randomised Controlled Trials

**DOI:** 10.1155/2018/7526234

**Published:** 2018-10-24

**Authors:** Dabin Lee, Hojung Lee, Jiwon Kim, Taehun Kim, Siyun Sung, Jungtae Leem, Tae-Hun Kim

**Affiliations:** ^1^College of Korean Medicine, Kyung Hee University, 26 Kyungheedae-ro, Dongdaemun-gu, Seoul 02447, Republic of Korea; ^2^Department of Clinical Korean Medicine, Graduate School, Kyung Hee University, 26 Kyungheedae-ro, Dongdaemun-gu, Seoul 02447, Republic of Korea; ^3^Korean Medicine Clinical Trial Center, Kyung Hee University Korean Medicine Hospital, 23 Kyungheedae-ro, Dongdaemun-gu, Seoul 02447, Republic of Korea; ^4^Chung-Yeon Medical Institute, 64, Sangmujungang-ro, Seo-gu, Gwangju 61949, Republic of Korea; ^5^Dongshin Korean Medicine Hospital, 351, Omok-ro, Yangcheon-gu, Seoul 07999, Republic of Korea

## Abstract

**Introduction:**

Infantile colic is a common condition causing considerable deterioration in the quality of life of both infants and their parents. Minimal acupuncture, a gentle needling technique without strong muscle stimulation, has primarily been used to treat this condition, but the clinical evidence of its efficacy and safety is yet to be established. The objective of this review was to assess clinical evidence of the safety and efficacy of acupuncture for infantile colic.

**Methods:**

To identify studies for inclusion, PubMed, Cochrane Library, Google Scholar, China Knowledge Resource Integrated Database, Wanfang, and Oriental Medicine Advanced Searching Integrated System were searched until January 2017. Only randomised controlled trials of infantile colic in patients aged 0 to 25 weeks, who were treated with acupuncture, were included. To assess the quality, the risk of bias was determined for each study by two authors. The intention was to perform a meta-analysis, but this was not possible in this study due to considerable clinical heterogeneity among the included studies.

**Results:**

Of the 601 studies identified, only four randomized controlled trials were included in this review. All included studies were conducted in northern European countries. Most studies showed a low risk of bias in most domains. Minimal acupuncture on LI4 or ST36 without strong stimulation was used in all studies. From the narrative analysis, acupuncture appears to be effective in alleviating the symptoms of colic, including crying and feeding and stooling problems, and may have only minor adverse effects. However, clinical evidence could not be confirmed owing to considerable clinical heterogeneity and the small sample sizes of the included studies.

**Conclusion:**

There is currently no conclusive evidence on the safety and efficacy of acupuncture for infantile colic. Rigorous full-scale randomized controlled trials will be necessary in future.

## 1. Introduction

Infantile colic is a common condition defined as colicky crying for more than 3 hours per day, more than 3 days per week, and longer than 3 weeks [1]. Infantile colic causes considerable deterioration of the quality of life of both infants and their parents. Colicky infants mainly suffer from crying, fussing, and paroxysms, sometimes with accompanying abdominal pain [2] or allergies [3]. In addition, parents of colicky infants may experience problems with physical and social functioning [4]. When considering the comparatively high incidence of infantile colic, ranging from 5% to 50% across different populations [5–7], the impact of infantile colic cannot be easily ignored.

Despite the diverse therapeutic strategies used worldwide, the efficacy of each intervention is still subject to dispute. In Europe, various treatments, including simethicone [8] and spinal manipulation [9], are known to be effective for reducing infantile crying. However, conflicting opinions on the efficacy of these interventions also exist [10]. Considering the lack of proven, effective treatments for infantile colic, alternative, and complementary treatments have come into the spotlight. Some studies note that herbal supplements have historically been used for stomach aches and colic [11]. Other studies suggest that acupuncture helps treat infantile colic [12] and reduces the pain and crying [13]. Acupuncture is a traditional East Asian medical treatment that is primarily defined as the insertion of needles at acupuncture points. Although its exact mechanism is not known, central and local analgesic effects can be achieved through stimulation with acupuncture [14]. Acupuncture is known to reduce pain associated with various diseases, including headaches in adults [15]. However, although there are various clinical studies available, there is no clinical evidence on the efficacy of acupuncture for treating infantile colic. These studies include a pilot study on ST36 acupuncture for infantile colic [16] and case studies on the effects of acupuncture treatment on gastrointestinal symptoms [17] and night crying [18]. Therefore, we conducted a systematic review (SR) of the effects of acupuncture on infantile colic using only randomised controlled trials (RCTs).

## 2. Methods

The primary objectives of this study were to assess clinical evidence of acupuncture for infantile colic compared with the control intervention. For assessing the clinical evidence on acupuncture for infantile colic, only RCTs were included in this study. Quasi-RCTs which adopted quasi-randomisation methods for the allocation of participants, such as alternation, were included in this study.

### 2.1. Participants

Only colicky infants aged 0 to 25 weeks were included. Based on the clinical definition of infantile colic, crying in early infancy had to total more than 3 hours per day and more than 3 days per week.

### 2.2. Intervention

All types of traditional acupuncture (including electroacupuncture), which consists of needle insertion at specific acupuncture points, were considered for inclusion. Nonpenetrating acupuncture point stimulation, including acupressure and laser acupuncture, was also included. If needles were inserted at nonclassical acupuncture points, the study was excluded.

### 2.3. Comparison

Conventional drug therapy, such as the use of an antifoaming agent to reduce excessive gas in the stomach or intestines, and no treatment were the comparative interventions in this study. If the acupuncture and control groups were treated with the same conventional treatments, the study was included. If there was a three-armed study which included different acupuncture intervention groups, the results of the different acupuncture intervention groups were intended to be combined and compared to the control group.

### 2.4. Outcomes

Diverse outcome measurements for total crying time, fussing, feeding, and stooling were analysed in this study. The definition of total crying time differed across the studies; therefore, the sum of the different levels of crying (fussing, crying, and colicky crying) times was regarded as the total crying time in some studies for the comparison. Outcomes for adverse events were also assessed.

### 2.5. Databases and Search Strategies

In this study, three core databases (Medline-PubMed [1990 to January 2017], Cochrane Library [1999 to January 2017], and Google Scholar [2002 to January 2017]), two Chinese databases (CNKI [China Knowledge Resource Integrated Database, Chinese Academic Journals, Conference Proceedings, and Theses; 1979 to January 2017] and Wanfang [1998 to January 2017]), and one Korean database (OASIS [Oriental Medicine Advanced Searching Integrated System]; 1963 to January 2017) were searched. There was no language limitation in this review. “Acupuncture” and “infantile colic” were used as the search terms for the development of the search strategy, which was modified based on the characteristics of each database (the appendix in the Supplementary Materials available here).

One author (HJL) conducted the literature searches and two authors (DBL and JWK) assessed the inclusion of the studies. Detailed information from each study was extracted by two authors independently using a predefined extraction form; differences in opinion were resolved through discussion. If there was any unclear information, we tried to contact the main authors of the studies. Acupuncture rationale, details of needling, treatment regimen, other components of treatment, practitioner background, controls, and comparator intervention were extracted based on the Standards for Reporting Interventions in Clinical Trials of Acupuncture (STRICTA) checklist [22].

### 2.6. Risk of Bias Assessment

The quality of the included studies was assessed using the risk of bias (ROB) tool from the Cochrane collaboration; each domain was graded as high risk (H), low risk (L), or unclear (U). Two authors (JWK and DBL) assessed each study and discussed the results. If there was any disagreement, a third person (HJL) made the final decision as an independent arbiter. The following seven domains were assessed: sequence generation, allocation concealment, blinding of participants and research personnel (acupuncture practitioners), blinding of outcome assessment, incomplete outcome data, selective outcome reporting, and other bias. In regard to the blinding of participants and research personnel, we felt that the ROB of all the studies could be graded as low because all the participants in the included studies were infants. In addition, the blinding of the personnel (acupuncture practitioners) was difficult due to the characteristics of the acupuncture itself. Therefore, we considered it impossible to achieve the perfect blinding of personnel for acupuncture. This did not significantly affect the results of the study and we graded blinding of the personnel as L. For selective reporting, we located the study protocol and graded it as L when there was no difference in the results between the protocol and the study results. When there was no protocol available, we graded it as L when all the important outcomes were reported in the study.

Since we expected that different outcome assessment tools would be used in most studies, we assessed effect estimates with a risk ratio (RR) for dichotomous outcomes and the standardized mean difference (SMD) for continuous outcomes. We had intended to perform a meta-analysis if enough studies had been included. If the clinical heterogeneity was not significant, a fixed-effect model analysis would be used for the meta-analysis and random-effect model analysis if the clinical heterogeneity was significant. If statistical and clinical heterogeneity were observed in the meta-analysis, a subgroup analysis would be conducted to evaluate possible reasons for heterogeneity. If a sufficient number of studies were included in the meta-analysis, we planned to use a funnel plot to visually assess publication bias.

## 3. Results

### 3.1. General Characteristics of the Included Studies

**Figure 1:**
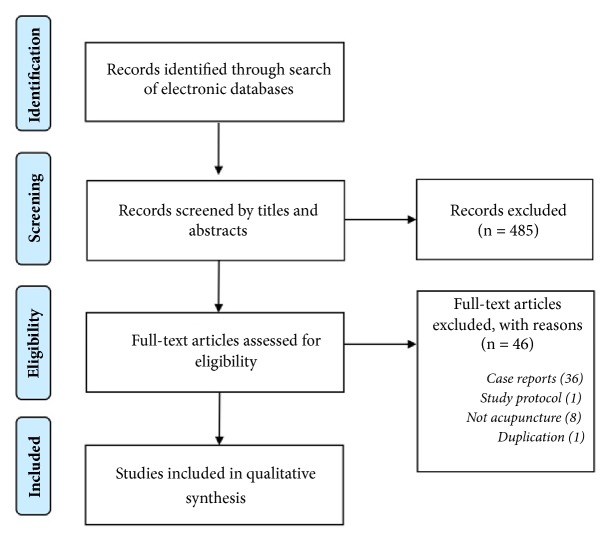
Study flow diagram.

As a result of the electronic database search, 601 studies were initially identified and four RCTs (a total of 357 infants) were included in this study (Figure 1). Participants in the four studies were aged 1 week to 25 weeks. Among the full-text articles that were reviewed, two were written by the same authors and involved identical baseline characteristics [19, 23]. We contacted the main authors and confirmed that the two studies could be regarded as one study [19].

Among the included studies, three were conducted in Sweden [13, 20, 21] and one was conducted in Norway [21]. Three were two-armed parallel group studies [13, 19, 21] and one included three groups (standardized minimal acupuncture, semistandardized acupuncture, and no-acupuncture groups) [20].

With regard to the assessment of the outcomes, the studies used different tools and questionnaires to assess the symptoms; thus performing a meta-analysis was not possible. Three studies adopted previously developed tools for outcome assessment [13, 19, 20] and one study did not provide any references for their outcome assessment tools [21]. In Landgren's study (2010) [19], parents reported 24 hours of fussing, crying, colicky crying, and total crying (TC) through use of a diary for least three days as the baseline and then every day during the three week of the intervention that was developed by Barr et al. [24] and Canivet et al. [25]. In addition, parents also reported adverse events through questionnaires developed by Reinthal et al. [13]. In the other study by Landgren (2016), researchers requested that parents report the severity of the symptoms of colic. [20]. Reinthal et al. used a 0-to-10 visual analogue scale (VAS) to assess the severity of infantile colic. A modified behavioural pain scale (MBPS) was used to assess the symptoms as a secondary outcome [13]. Three studies involved follow-up assessments, while one study performed assessments during the treatment period only [19]. Skjeie et al. assessed participants during the 4-week treatment period, as well as after the treatment [21]. Reinthal et al. evaluated infants 1 week after treatment [13]. Landgren et al. (2016) assessed infants in the first and second weeks of treatment and then three days after the completion of the 2-week treatment course (Table 1) [20].

With regard to the details of the acupuncture procedures, one study [13] stimulated the LI4 point on both the left and right hands, while two studies [19, 20] alternated the stimulation between the LI4 points. Another study offered acupuncture at ST36 on both left and right legs [21]. In one study, needles were inserted and withdrawn at LI4 without stimulation in one group and a maximum of five-point insertions among Sifeng, LI4, and ST36 were selected based on a patient's symptoms; the needles were retained for a maximum 30 seconds in the other intervention group [20]. Minimal acupuncture without manual stimulation was offered to infants in three studies [19–21] and manual stimulation including rotating needles was performed in one study [13]. The number of treatment sessions ranged from 3 to 6 times over 3 days to 3 weeks (Table 2).

### 3.2. Risk of Bias Assessment

Most studies showed a low ROB in all domains, with the exception of one study [13]. Selective outcome reporting and incomplete outcome data domains showed low ROB in all the included studies. Although no study adopted sham acupuncture needles or sham procedures as control interventions, the blinding of participants was successful because the study population in this review consisted entirely of infants and outcomes were assessed through parental reporting; the parents did not know if their babies had been treated with acupuncture [19–21].

### 3.3. Total Crying Time

Three studies examined the total crying time (min/day) in colicky infants [19–21]. Landgren et al. (2010) measured the total duration of crying (min/day) during the three weeks of intervention (6 acupuncture sessions) and found a significant difference in the median crying duration between the groups during the first (acupuncture group: 193 min/day [interquartile range, IQR: 143–253 min/day] and control group: 225 min/day [IQR: 178–316 min/day]; p=0.025) and second (acupuncture group: 164 min/day [IQR: 103 – 201 min/day] and control group: 188 min/day [IQR: 149–273 min/day]; p=0.016) weeks, but not the third intervention week (acupuncture group: 149 min/day [IQR: 119 – 267 min/day] and control group: 169 min/day [IQR: 119–267 min/day]; p=0.062) [19].

Skjeie et al. examined the crying minutes per day during the four weeks after three acupuncture sessions. On the third intervention day, the average total crying time was 129 min (95% CI: 97, 162) in the acupuncture group and 167 min (95% CI: 123, 211) in the control group; the difference was -38 min (95% CI: -91, 17). Four weeks after acupuncture treatment, the average crying time was 89 min (95% CI: 55, 123) in the acupuncture group and 97 min (95% CI: 67, 126) in the control group; the difference was -8 min (95% CI: -51, 37). The total cry time decreased, but the differences were not statistically significant on the third intervention day (p=0.17) or 4 weeks after treatment with acupuncture (p=0.74) [21].

Landgren et al. (2016) evaluated the total crying time during the six days after two weeks of acupuncture (4 acupuncture sessions) and found that there was a significant difference in the median total crying duration between the acupuncture and control groups in the first (acupuncture group: 170 min/day [IQR: 132–236 min/day] and control group: 206 min/day [IQR: 153–270 min/day]; p=0.032) and the second (acupuncture group: 137 min/day [IQR: 101–200 min/day] and control group: 176 min/day [IQR: 133–223 min/day]; p=0.020) weeks of intervention. However, there was no difference six days after two weeks of acupuncture treatment (acupuncture group: 123 min/day [IQR: 87–197 min/day] and control group: 164 min/day [IQR: 112–230 min/day]; p=0.073) [20].

Reinthal et al. did not include a total crying time, but they showed individual crying times for each quarter of the day. They found that the median crying duration decreased from midnight to 6:00 am (acupuncture group: 0 min [minimum: 0 to maximum: 10] and control group: 0 min [minimum: 0 to maximum: 120]) and from 6:00 pm to midnight (acupuncture group: 20 min [minimum: 0 to maximum: 120] and control group: 31 min [minimum: 0 to maximum: 120]) in both groups after treatment with acupuncture [13].

### 3.4. Colicky Crying

Two studies assessed the colicky crying time (min/day). Landgren et al. (2000) measured the total duration of colicky crying at baseline and during the first, second, and third weeks of intervention. During the second week of intervention, the median duration of colicky crying was 9 min/day [IQR: 0–27 min/day] in the treatment group and 13 min/day [IQR: 4-49 min/day] in the control group (p=0.046). Colicky crying time was also shorter in the treatment group during the first and third weeks, but there was no statistically significant difference [19].

The other study by Landgren (2016) compared the total duration of colicky crying at baseline and during the interventional and the follow-up periods. In the infants who received either acupuncture treatments, standardized, or individualized acupuncture (n=98), the median colicky crying time decreased from 42 min/day [IQR: 17-92 min/day] to 3 min/day [IQR: 0-17 min/day] at the follow-up visit. In the control group (n=49), the median time of colicky crying decreased from 55 min/day [IQR: 22-101 min/day] to 13 min/day [IQR: 0-26 min/day] in the follow-up visit. Total duration of colicky crying at follow-up was significantly different between the acupuncture and control groups [20].

### 3.5. Fussing

Two studies examined the duration of fussing (min/day) in colicky infants [19, 20]. After acupuncture treatment sessions, median fussing time per day reportedly decreased, but there was no significant difference between the groups (69 min/day [IQR: 36–103 min/day] in the acupuncture group and 85 min/day [IQR: 63–151 min/day] in the control group; p=0.119) [19]. These results were similar to those of another study. The total fussing time per day was reported to decrease after treatment with acupuncture, but there was no significant difference between the groups (66 min/day [IQR: 41–100] in the acupuncture group and 88 min/day [IQR: 45–133] in the control group; p=0.0.173) [20].

### 3.6. Feeding

One study examined the feeding times. Landgren et al. compared the feeding times (min/day) using the Mann-Whitney* U* test at baseline and during the intervention weeks. The median feeding time at baseline was 155 min/day [IQR: 113–193] for the treatment group and 140 min/day [IQR: 118–178] for the control group. Median feeding time during the third intervention week was 140 min/day [IQR: 108–196)] for the treatment group and 145 min/day [IQR: 104–188] for the control group. Feeding time in the control group after intervention was slightly longer than that of the treatment group, but it was not statistically significant (p=0.854) [23].

### 3.7. Stooling

One study analysed the stooling frequency (times/day) and stooling patterns in colicky infants. Landgren et al. compared the daily stooling times at baseline and during the intervention weeks using the Mann-Whitney* U* test. The median baseline stooling frequency of the treatment group was 4.1 times/day [IQR: 2.2-6.0] and was 4.3 times/day [IQR: 2.7-6.5] in the control group. During the third intervention week, the median stooling frequency was 2.1 times/day [IQR: 1.1-4.7] for the treatment group and 3.1 times/day [IQR: 1.0-4.6] for the control group. Stooling frequency decreased considerably in the treatment group over baseline, but it was not statistically significant (p=0.902). Stooling patterns were also assessed by the parents. Almost twice as many control group parents described stools as “more watery” and almost three times as many treatment group parents described stools as “more firm” or “normalized” [23].

### 3.8. Adverse Events

Among the four studies, three studies referred to adverse events, while one study did not mention any adverse events [13]. In the 2010 study by Landgren et al., slight bleeding (one drop) of blood was detected after acupuncture in one patient. In addition, 74% of participants in the treatment group cried for more than 10 seconds after treatment, 14% of the infants cried for more than 1 min, and no infants cried for more than 2 min [19]. Possible adverse effects were also collected from the parents' questionnaire results. Three infants in the treatment group became more restless after the visit, while no subject in the control group exhibited the same behaviour. One infant in the treatment group wanted to eat more often, while no subject in the control group exhibited the same behaviour. Two cases of increased skin symptoms were reported in the treatment group, while one case was reported in the control group [19]. Skjeie et al. reported that there were no serious side effects [21]. The other Landgren study (2016) suggested that among the 388 acupuncture sessions, infants cried up to 1 min on 157 occasions and for more than 1 min on 31 occasions. Fifteen cases of a single drop of blood were reported after acupuncture, a drop of blood was found on the infant's clothes in one case, and a mark on the hand was reported in another case [20].

### 3.9. Allergy

Among the four studies, one study mentioned allergies, while three studies did not. Landgren et al. (2010) found that 17% of the acupuncture group and 18% of the control group had a family history of allergies. Parents were asked to exclude cow milk proteins during the baseline registration to keep milk protein allergies to a minimum; those who stopped crying were not included in the study [19].

## 4. Discussion

Among the 601 identified studies, four RCTs (a total of 357 infants) were included in this review; three of these were RCTs [19–21] and one study was a quasi-RCT [13]. Most studies had a low ROB in all domains except the outcome of the assessor blinding. Included studies used minimal acupuncture at LI4, ST36, and Sifeng without strong stimulation. Because different outcome assessment tools were used by different studies, a quantitative meta-analysis on the safety and efficacy associated with acupuncture treatment could not be performed. From the narrative analysis of the included studies, we found that acupuncture may be effective for reducing the symptoms of colic, including crying, feeding, and stooling problems and may be associated with only minor adverse events. However, none of the studies recruited a sufficient number of patients and there was considerable clinical heterogeneity in the study designs and outcome assessment tools. Therefore, the clinical evidence regarding the effects of acupuncture on infantile colic is inconclusive.

This study has several strong points. First, we applied a systematic electronic database search strategy. In addition to the core databases (Medline and the Cochrane Library), we assessed local Korean and Chinese databases in order to reduce publication bias, although, ultimately, there were no RCTs conducted in these countries. Second, if there were unclear points in the literature, we contacted the authors of the original studies to collect accurate information. Through this effort, we were able to exclude duplicate studies. In addition, the details of the study designs, including the outcome assessment methods, were obtained directly from the authors if necessary. All steps in this SR are recommended as standard protocol, but they can be easily ignored [26]. Third, considering that the study population comprised infants for whom classic needle acupuncture was not feasible in some cases, we adopted a broader definition of acupuncture intervention; acupressure and laser acupuncture, which are nonpenetrating acupuncture point stimulation methods, were also included in the search strategy. Although there were no acupressure or laser acupuncture studies included in this review, we made a meaningful attempt to include this population.

Limitations of this study should also be described here. The first and most important limitation is that conclusive evidence on whether acupuncture is an effective intervention for infantile colic cannot be determined from this review due to the limited number of original studies. Summary effect estimates could not be calculated in this study. In addition, all included RCTs were small studies that could not ensure sufficient statistical power to assess the effects of acupuncture for this condition. Meta-analysis is a meaningful statistical method when there are clinical questions that are not solved through individual studies [26]. However, if there is significant heterogeneity in the study populations, interventions, outcome assessment tools, time points, and the interventions being compared, reliability of the meta-analysis results decreases significantly. In this review, we could only extract clinical outcomes from the four identified RCTs in various aspects of colic individually and analyse them in a narrative manner because the studies did not all adopt common outcome assessment tools. Second, only a small number of RCTs were available for this review. Even though we adopted an extensive search strategy of the core and local medical databases without language limitations, only four studies were identified for this review. The small number of RCTs is one of the major reasons for the inconclusive evidence on the effectiveness of acupuncture treatment for infantile colic. Third, the inclusion criteria for this review were slightly relaxed. Quasi-RCTs were included in this review. Participants aged between 0 and 25 weeks were included even though, in most infants, the symptoms of colic spontaneously disappear within 3 months after birth [1]. This slightly relaxed strategy for literature selection made it possible to include more studies but reduced the reliability of the results and they needed to be interpreted with caution.

One item we need to discuss further is the reason that all studies were conducted in Scandinavian countries. An SR on observational studies for infants suggested that the frequency of infantile colic is up to 40% among Caucasian infants [5]. The frequency may be lower in the Asian populations, since one study observed no infantile colic among 160 Korean infants [27]. There is no concrete evidence on whether ethnicity is a potential risk factor for infantile colic. However, diet, feeding methods, and the practice of the care of infants are different across the different areas and cultures; this may affect the incidence of infantile colic. In addition, there could be cultural differences in the acceptance of an infant crying. The fact that infantile colic may not be seen as a serious problem in Asian countries is one possible explanation for why no clinical trials have been conducted in Asian countries, even though the practice of acupuncture has been actively used to treat other conditions.

Another issue is the correlation between diet and the symptoms of colic. There has been debates on the causal relationship between food allergy and infantile colic. In addition to this, dysmotility related to the hypersensitivity and dysbiosis is presumed to be a possible cause of infantile colic [28]. From the result of the Landgren study (2016), about 36% of the initial infants screened showed an improvement after restricting cow's milk protein from the diet for at least 5 days; they were excluded from the study, which suggests that diet could affect the symptoms of colic in many infants [20]. However, participants of this study (about 36% of the infants) still fulfilled the criteria for colic after diet control, which implies that factors other than diet are also responsible for infantile colic.

Considering that pathogenesis of infantile colic is not fully defined, the mechanism of action for acupuncture is not expected to be simply explained either. Several hypotheses for infantile colic, including immaturity of the nervous and/or digestive system, cow's milk protein allergy, altered gut normal flora, and abnormal gut hormone levels in infants have been suggested. In conventional medicine, the administration of an antifoaming agent, simethicone, and pain relieving agents is considered the standard treatments, in addition to avoiding a high allergen diet for breast-feeding mothers and specialized dietary formula for bottle-fed infants [29]. In addition to this, several behaviour therapies including car ride stimulation, counselling, modified parent-child interaction, and contingent music have been used for infantile colic as an alternative treatment strategy. For example, there is a clinical report about continuous vibroacoustic stimulation which might be effective for reducing colic symptoms [30]. However, there are still debates on the clinical effectiveness of these behaviour interventions [31]. Acupuncture at LI4 or ST36 seems to reduce excessive GI excitability and pain. In regard to increased gastrointestinal (GI) tract contractions due to GI hormonal changes and absorption problems in the GI tract, such as lactose intolerance [29], results from rat studies show that acupuncture at ST36 might inhibit colon-transit acceleration induced by stress [32] and decrease visceral hypersensitivity and enhanced excitability in the colon via endogenous opiate signalling pathways [33]. Acupuncture at LI4 might contribute to the inhibition of over-active GI peristalsis via sympathetic nerve stimulation of the median nerve [34]. However, there are several issues regarding acupuncture technique that warrant discussion. Differences in intensity, frequency, depth, retention time, and stimulation methods of acupuncture treatment might exert different therapeutic effects. For instance, the effect of acupuncture can include a very fast analgesic effect as well as a slowly improving therapeutic effect; however, the four studies included in this review only adopted 4 to 6 sessions of short-term acupuncture, which could not confirm the long-term therapeutic effect of acupuncture induced after weeks of successive treatment sessions [35]. In addition, most experimental studies on the analgesic effect of acupuncture adopt electroacupuncture, which includes high intensity stimulation, but studies of this review only used manual minimal acupuncture, which only included week stimulation [35]. Future studies need to evaluate the various features of acupuncture techniques, which might have variable effects on short-term and long-term outcomes, as well as diverse symptoms of infantile colic.

### 4.1. Conclusions

There is no conclusive evidence on the safety and efficacy of the use of acupuncture to treat infantile colic. All the included studies had a small number of participants and used different acupuncture points, insertion times, and needling methods, which potentially contributed to the inclusive evidence of acupuncture for this condition. Future clinical studies need to adopt common validated instruments with confirmed reliability and validity. In addition, better information on the mechanism action of acupuncture in infantile colic is needed to better understand the use of this treatment.

## Figures and Tables

**Table 1:** Characteristics of included studies.

**Study ID**	**Age range (week)**	**Country**	**Maternal history (%)**	**Acupuncture group** **(n)**	**Control group** **(n)**	**Main outcomes**	**Risk of bias assessment**						**Conclusion** **∗**
							**1**	**2**	**3**	**4**	**5**	**6**	
**Landgren 2010 [19]**	2-8	Sweden	(i) Acupuncture group: food intolerance/allergy (37%), had infantile colic (45%) (ii) Control group: food intolerance/allergy (63%), had infantile colic (58%)	Standardized manual acupuncture (46)	No treatment (40)	(i)Duration of fussing, crying and colicky crying (ii) Adverse events	L	L	L	L	L	L	“Standardised, light stimulation of the acupuncture point LI4 twice a week for 3 weeks reduced the duration and intensity of crying more quickly in the acupuncture group than in the control group.”
**Landgren 2016 [20]**	2-8	Sweden	Not reported	A: Standardized manual acupuncture (49) + usual care with nurse consultation B: Semi-standardized acupuncture (49) ) + usual care with nurse consultation	Usual care with nurse consultation (49)	(i) Total crying time (ii) Number of colic infants (iii) Adverse events	L	L	L	L	L	L	“Minimal acupuncture shortened the duration and reduced the intensity of crying in infants with colic.”
**Reinthal 2008 [13]**	1-25	Sweden	Not reported	Standardized manual acupuncture + simethicone solution (20)	Simethicone solution (20)	(i) Crying per day (ii) Modified Behavioral Pain Scale(MBPS)	U	U	U	U	L	L	“Four treatments with light needing on one point in the hand may alleviate crying and pain related behavior without any noted side effects.”
**Skjeie 2013 [21]**	3-13	Norway	Not reported	Standardized manual acupuncture (44)	No treatment (40)	(i) Minutes of crying per day (ii) General assessment of the child's condition (iii) Adverse effects.	L	L	L	L	L	L	“This trial of acupuncture treatment for infantile colic showed no statistically significant or clinically relevant effect.”

1: sequence generation; 2: allocation concealment; 3: blinding of participants; 4: blinding of outcome assessor; 5: selective reporting; 6: incomplete outcome; low risk of bias: L; high risk of bias: H; unclear risk of bias: U. *∗*Conclusion was extracted from the published article.

**Table 2:** Details of acupuncture treatment.

**Study ID (author, year)**	**(1) Acupuncture rationale**	**(2) Details of needling**	**(3) Treatment regimen**	**(4) Other components **	**(5) Practitioner background**	**(6) Control intervention**
	*(1a) Style* *(1b) Reasoning basis* *(1c) Extent of treatment variability*	*(2a) Number / (2b) Points* *(2c) Depth / (2d) Response* *(2e) Stimulation / (2f) Retention* *(2g) Type*	*(3a) Sessions* *(3b) Frequency and duration*	*(4a) Details* *(4b) Context*	*(5a) Description*	*(6a) Rationale* *(6b) Comparator intervention*
Landgren 2010 [19]	(1a) Minimal, standardized acupuncture (1b) Not mentioned (1c) Not allowed	(2a) 2 points / (2b) Unilateral LI4 (2c) 2mm / (2d) Not mentioned (2e) Without manipulation / (2f) 2s (2g) Disposable sterile stainless needles, 0.20*∗*13mm	(3a) 6 times (3b) 3 weeks, 2 times/week	(4a) Not mentioned (4b) The infant was treated by a nurse who was alone with the baby in the treatment room.	(5a) Nurse skilled in acupuncture	(6b) No treatment
Landgren 2016 [20]	(1a) Group A: Standardized, minimal acupuncture Group B: Semi-standardized individual acupuncture (1b) Traditional Chinese medicine (1c) Group A: Not allowed Group B: Semi-individual acupuncture	(2a) Group A: 1 point Group B: up to 5 points (2b) Group A=LI4 Group B= choose one point, or any combination of Sifeng, LI4 and ST36, depending on the infant's symptoms (2c) Group A=3mm Group B=1mm for Sifeng, 3mm for LI4, ST36 (2d) No deqi in both groups (2e) Group A=withdrawn with stimulation, unilaterally Group B=minimal stimulation, uni- or bilaterally (2f) Group A=2-5s Group B=1s for Sifeng, up to 30 sec for LI4 and ST36 (2g) Disposable sterile stainless needles, 0.20*∗*13mm	(3a) 4 times (3b) 2 weeks, 2 times/week	(4a) Not mentioned (4b) For all groups, it is encouraged to keep breastfeeding for breastfeeding mothers. All children received usual care and four extra consultations, which is called gold standard care. The infant was treated by an acupuncture practitioner who was alone with the baby in the treatment room.	(5a) 10 acupuncturists who had undergone acupuncture practice for a mean duration of 20 years	(6b) Usual care and four extra consultations
Reinthal 2008 [13]	(1a) Minimal acupuncture (light needling) (1b) Not mentioned (1c) Not allowed	(2a) 2 points / (2b) Both LI4 (2c) Deep enough to reach the dorsal interosseous muscle / (2d) Rotated (2e) Rotated, left, then removed / (2f) 2-5s (2g) Disposable sterile stainless needles, 0.2mm	(3a) 4 times (3b) 2 weeks, 2 times/week	(4a) Simethicone (4b) For all groups, simethicone was offered. The infant was treated by a midwife who was alone with the baby in the treatment room.	(5a) Midwife trained in Western acupuncture and practicing it for more than 15 years	(6b) No treatment
Skjeie 2013 [21]	(1a) Standardized acupuncture (1b) Not mentioned (1c) Not allowed	(2a) 2 points / (2b) Both ST36 (2c) 12mm / (2d) Not mentioned (2e) Without manipulation / (2f) 30s (2g) Disposable sterile stainless needles, 0.20*∗*15mm	(3a) 3 times (3b) 3 consecutive days, 1 time/day	(4a) Not mentioned (4b) The GP was alone in the treatment room with the baby in the treatment room.	(5a) GPs educated from the programs of the Norwegian Society of Medical Acupuncture	(6b) No treatment

GP: general practitioner.

## Supplementary Materials

Supplementary Materials Appendix 1. Searching strategy: Detailed searching strategies for Medline, CNKI, Wangfang, OASIS, Google scholar and Cochrane library.
