# Supplementary material for: Acupuncture for Infantile Colic: A Systematic Review of Randomised Controlled Trials
Source: Evid Based Complement Alternat Med. 2018 Oct 24;2018:7526234. doi: 10.1155/2018/7526234 (PMC6220386; doi:10.1155/2018/7526234)
Supplement: Supplementary Materials — Appendix 1. Searching strategy: Detailed searching strategies for Medline, CNKI, Wangfang, OASIS, Google scholar and Cochrane library. [file 7526234.f1.pdf]

Appendix 1. Searching strategy

Medline-PubMed

#1. acupuncture
#2. acupressure
#3. acupoint
#4. #1 OR #2 OR #3
#5. electro-acupuncture
#6. meridian
#7. electroacupuncture
#8. moxibust
#9. traditional chinese medicine
#10. traditional oriental medicine
#11. ACUPUNCTURE THERAPY
#12. ACUPUNCTURE[MeSH Terms]
#13. #5 OR #6 OR #7 OR #8 OR #9 OR #10 OR #11 OR #12
#14. #4 OR #13
#15. stomach
#16. abdominal
#17. abdomen
#18. spasm
#19. pain
#20. cramp
#21. #15 OR #16 OR #17 OR #18 OR #19 OR #20
#22. colic[Title/Abstract]
#23. colic[MeSH Terms]))
#24. #21 OR #22 OR #23
#25. crying[Title/Abstract]
#26. cry[Title/Abstract]
#27. cries[Title/Abstract]
#28. #25 OR #26 OR #27
#29. infants
#30. newborn
#31. Baby
#32. babies
#33. children
#34. #29 OR # 30 OR #31 OR #32 OR #33
#35. #28 AND #34
#36. #21 AND #35
#37. #14 AND #36

CNKI and Wanfang

#1. 针

#2. 针灸

#3. 耳针

#4. 耳针

#5. 电针

#6. 激光针

#7. #1 OR #2 OR #3 OR #4 OR #5 OR #6

#8. 腹痛

#9. 疝气

#10. 疝痛

#11. 夜啼

#12. #8 OR #9 OR #10 OR #11

#13. 小儿

#14. 婴儿

#15. #13 OR #14

#16. #7 AND #12 AND #15

OASIS, Google Scholar and Cochrane Library

#1. acupuncture
#2. acupuncture
#3. infantile
#4. #1 AND #2 AND #3
